# Nanotechnology-based drug delivery systems

**DOI:** 10.1186/1745-6673-2-16

**Published:** 2007-12-01

**Authors:** Sarabjeet Singh Suri, Hicham Fenniri, Baljit Singh

**Affiliations:** 1 Department of Veterinary Biomedical Sciences and Immunology Research Group, University of Saskatchewan, 52 Campus Drive, Saskatoon, SK, S7N 5B4, Canada; 2 National Institute of Nanotechnology, National Research Council (NINT-NRC) and Department of Chemistry, University of Alberta, 11421 Saskatchewan Drive, Edmonton, AB, T6G 2M9, Canada

## Abstract

Nanoparticles hold tremendous potential as an effective drug delivery system. In this review we discussed recent developments in nanotechnology for drug delivery. To overcome the problems of gene and drug delivery, nanotechnology has gained interest in recent years. Nanosystems with different compositions and biological properties have been extensively investigated for drug and gene delivery applications. To achieve efficient drug delivery it is important to understand the interactions of nanomaterials with the biological environment, targeting cell-surface receptors, drug release, multiple drug administration, stability of therapeutic agents and molecular mechanisms of cell signalling involved in pathobiology of the disease under consideration. Several anti-cancer drugs including paclitaxel, doxorubicin, 5-fluorouracil and dexamethasone have been successfully formulated using nanomaterials. Quantom dots, chitosan, Polylactic/glycolic acid (PLGA) and PLGA-based nanoparticles have also been used for *in vitro *RNAi delivery. Brain cancer is one of the most difficult malignancies to detect and treat mainly because of the difficulty in getting imaging and therapeutic agents past the blood-brain barrier and into the brain. Anti-cancer drugs such as loperamide and doxorubicin bound to nanomaterials have been shown to cross the intact blood-brain barrier and released at therapeutic concentrations in the brain. The use of nanomaterials including peptide-based nanotubes to target the vascular endothelial growth factor (VEGF) receptor and cell adhesion molecules like integrins, cadherins and selectins, is a new approach to control disease progression.

## Introduction

Nanoparticles used as drug delivery vehicles are generally < 100 nm in at least one dimension, and consist of different biodegradable materials such as natural or synthetic polymers, lipids, or metals. Nanoparticles are taken up by cells more efficiently than larger micromolecules and therefore, could be used as effective transport and delivery systems. For therapeutic applications, drugs can either be integrated in the matrix of the particle or attached to the particle surface. A drug targeting system should be able to control the fate of a drug entering the biological environment. Nanosystems with different compositions and biological properties have been extensively investigated for drug and gene delivery applications [1-5]. An effective approach for achieving efficient drug delivery would be to rationally develop nanosystems based on the understanding of their interactions with the biological environment, target cell population, target cell-surface receptors [6], changes in cell receptors that occur with progression of disease, mechanism and site of drug action, drug retention, multiple drug administration, molecular mechanisms, and pathobiology of the disease under consideration. It is also important to understand the barriers to drug such as stability of therapeutic agents in the living cell environment. Reduced drug efficacy could be due to instability of drug inside the cell, unavailability due to multiple targeting or chemical properties of delivering molecules, alterations in genetic makeup of cell-surface receptors, over-expression of efflux pumps, changes in signalling pathways with the progression of disease, or drug degradation. For instance, excessive DNA methylation with the progression of cancer [7] causes failure of several anti-neoplastic agents like doxorubicin and cisplatin. Better understanding of the mechanism of uptake, intracellular trafficking, retention, and protection from degradation inside a cell are required for enhancing efficacy of the encapsulated therapeutic agent.

In this review we discuss the drug delivery aspects of nanomedicine, the molecular mechanisms underlying the interactions of nanoparticles with cell-surface receptors, biological responses and cell signalling, and the research needed for the widespread application of nanodelivery systems in medicine.

## Design of nanotechnology – based drug delivery Systems

**Figure 1:**
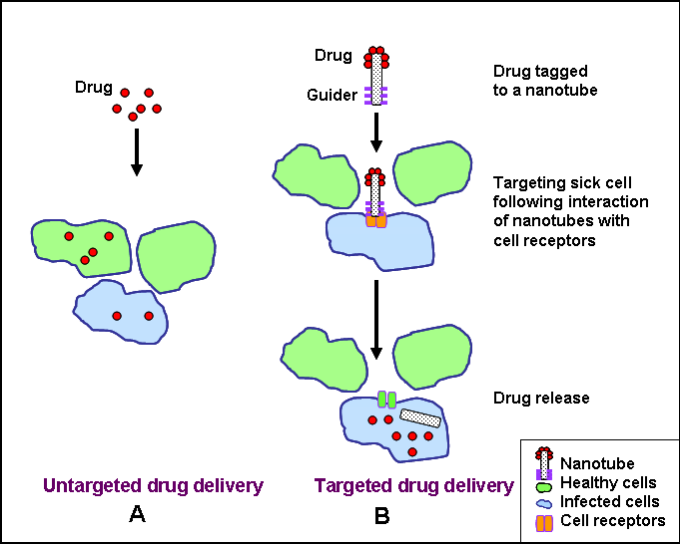


Nanoparticles can be used in targeted drug delivery at the site of disease to improve the uptake of poorly soluble drugs [8,9], the targeting of drugs to a specific site, and drug bioavailability. A schematic comparison of untargeted and targeted drug delivery systems is shown in Figure 1. Several anti-cancer drugs including paclitaxel [10,11], doxorubicin [12], 5-fluorouracil [13] and dexamethasone [14] have been successfully formulated using nanomaterials. Polylactic/glycolic acid (PLGA) and polylactic acid (PLA) based nanoparticles have been formulated to encapsulate dexamethasone, a glucocorticoid with an intracellular site of action. Dexamethasone is a chemotherapeutic agent that has anti-proliferative and anti-inflammatory effects. The drug binds to the cytoplasmic receptors and the subsequent drug-receptor complex is transported to the nucleus resulting in the expression of certain genes that control cell proliferation [14]. These drug-loaded nanoparticles formulations that release higher doses of drug for prolonged period of time completely inhibited proliferation of vascular smooth muscle cells.

Colloidal drug delivery modalities such as liposomes, micelles or nanoparticles have been intensively investigated for their use in cancer therapy. The effectiveness of drug delivery systems can be attributed to their small size, reduced drug toxicity, controlled time release of the drug and modification of drug pharmacokinetics and biological distribution. Too often, chemotherapy fails to cure cancer because some tumor cells develop resistance to multiple anticancer drugs. In most cases, resistance develops when cancer cells begin expressing a protein, known as p-glycoprotein that is capable of pumping anticancer drugs out of a cell as quickly as they cross through the cell's outer membrane. New research shows that nanoparticles may be able to get anticancer drugs into cells without triggering the p-glycoprotein pump [11,15]. The researchers studied *in vivo *efficacy of paclitaxel loaded nanoparticles in paclitaxel-resistant human colorectal tumors. Paclitaxel entrapped in emulsifying wax nanoparticles was shown to overcome drug resistance in a human colon adenocarcinoma cell line (HCT-15). The insolubility problems encountered with paclitaxel can be overcome by conjugating this drug with albumin. Paclitaxel bound to bio-compatible proteins like albumin (Abraxane) is an injectable nano-suspension approved for the treatment of breast cancer. The solvent Cremophor-EL used in previous formulations of paclitaxel causes acute hypersensitivity reactions. To reduce the risk of allergic reactions when receiving paclitaxel, patients must undergo pre-medication using steroids and anti-histamines and be given the drug using slow infusions lasting a few hours. Binding paclitaxel to albumin resulted in delivery of higher dose of drug in short period of time. Because it is solvent-free, solvent-related toxicities are also eliminated. In Phase III clinical trial, the response rate of Abraxane was about twice than that of the solvent-containing drug Taxol.

## Nanoparticle-mediated delivery of siRNA

Short interfering RNA (siRNA) is emerging as a robust method of controlling gene expression with a large number of applications. Translation of nucleic acid-based therapy to clinical studies will require significant advances in the delivery system. Quantum dots (QD) have been used to monitor RNAi delivery [16]. PLGA and PLA based nanoparticles have also been used for *in vitro *RNAi delivery [17]. Although there has been some success in the delivery of siRNA using various nanomaterials, tracking their delivery and monitoring their transfection efficiency is difficult without a suitable tracking agent or marker. Designing an efficient and self-tracking transfection agent for RNA interference is a big challenge. Recently, Tan *et al *[18] synthesized chitosan nanoparticles encapsulated with quantum dots and used such nanomaterial to deliver human epidermal growth factor receptor-2 (HER2/neu) siRNA. Such a novel nano carrier helped in monitoring the siRNA by the presence of fluorescent QDs in the chitosan nanoparticles. Targeted delivery of HER2 siRNA to HER2-overexpressing SKBR3 breast cancer cells has been specific with chitosan/quantum dot nanoparticles surface labeled with HER2 antibody targeting the HER2 receptors on SKBR3 cells [18].

Labeling of nanoparticles with a fluorescent marker, such as Cy-5, helps in visualizing uptake and accumulation of nanotubes using a fluorescent microscope. Recently, Howard *et al *[19] used such nanoparticles conjugated with siRNA specific to the BCR/ABL-1 junction sequence and found 90% reduced expression of BCR/ABL-1 leukemia fusion protein in K562 (Ph(+)) cells. Effective *in vivo *RNA interference was also achieved in bronchiolar epithelial cells of transgenic EGFP mice after nasal administration of chitosan/siRNA formulations. These findings highlight the potential application of this novel chitosan-based system in RNA-mediated therapy of systemic and mucosal disease.

## Cancer

### Targeting cancer cells with nanoparticles

Cancer is one of the most challenging diseases today, and brain cancer is one of the most difficult malignancies to detect and treat mainly because of the difficulty in getting imaging and therapeutic agents across the blood-brain barrier and into the brain. Many investigators have found that nanoparticles hold promise for ferrying such agents into the brain [20-22]. Apolipoprotein E was suggested to mediate drug transport across the blood-brain barrier [23]. Loperamide, which does not cross the blood-brain barrier but exerts antinociceptive effects after direct injection into the brain, was loaded into human serum albumin nanoparticles and linked to apolipoprotein E. Mice treated intravenously with this complex induced antinociceptive effects in the tail-flick test. The efficacy of this drug delivery system of course depends upon the recognition of lipoprotein receptors. Kopelman and colleagues designed Probes Encapsulated by Biologically Localized Embedding (PEBBLE) to carry a variety of unique agents on their surface and to perform multiple functions [22]. One target molecule immobilized on the surface could guide the PEBBLE to a tumor. Another agent could be used to help visualize the target using magnetic resonance imaging, while a third agent attached to the PEBBLE could deliver a destructive dose of drug or toxin to nearby cancer cells. All three functions can be combined in a single tiny polymer sphere to make a potent weapon against cancer. Another anti-cancer drug, doxorubicin, bound to polysorbate-coated nanoparticles is able to cross the intact blood-brain barrier and be released at therapeutic concentrations in the brain [24]. Smart superparamagnetic iron oxide particle conjugates can be used to target and locate brain tumors earlier and more accurately than reported methods [25]. It is known that folic acid combined with polyethylene glycol can further enhance the targeting and intracellular uptake of the nanoparticles. Therefore, nanomaterial holds tremendous potential as a carrier for drugs to target cancer cells.

### Targeting angiogenesis with nanoparticles

Robust angiogenesis underlies aggressive growth of tumors. Therefore, one of the mechanisms to inhibit angiogenesis is to starve tumor cells. Angiogenesis is regulated through a complex set of mediators and recent evidence shows that integrin αvβ3 and vascular endothelial growth factors (VEGFs) play important regulator roles. Therefore, selective targeting of αvβ3 integrin and VEGFs is a novel anti-angiogenesis strategy for treating a wide variety of solid tumors. One approach is to coat nanoparticles with peptides that bind specifically to the αvβ3 integrin and the VEGF receptor [26]. The synthetic peptide bearing Arg-Gly-Asp (RGD) sequence is known to specifically bind to the αvβ3 integrin expressed on endothelial cells in the angiogenic blood vessels, which can potentially inhibit the tumor growth and proliferation. Following hydrophobic modifications, glycol chitosan is capable of forming self-aggregated nanotube and has been used as a carrier for the RGD peptide, labeled with fluoresein isothiocyanate (FITC-GRGDS) [27]. These nanotubes loaded with FITC-GRGDS might be useful for monitoring or destroying the angiogenic tissue/blood vessels surrounding the tumor tissue. Our research group has been studying biological responses of RGDSK self-assembling rosette nanotubes (RGDSK-RNT). These rosette nanotubes are a novel class of nanotubes that are biologically inspired and naturally water soluble upon synthesis [28,29]. These nanotubes are formed from guanine-cytosine motif as building blocks. However, one of the novel properties of the RNT is the ability to accept a variety of functional groups at the G/C motif which imparts functional versatility to the nanotubes for specific medical or biological applications. Therefore, the RNTs can be potentially modified to target a variety of therapeutic molecules in vivo to treat cancer and inflammatory diseases.

## Nanosystems in inflammation

### Targeting macrophages to control inflammation

The potential of macrophages for rapid recognition and clearance of foreign particles has provided a rational approach to macrophage-specific targeting with nanoparticles. Macrophages' ability to secrete a multitude of inflammatory mediators allows them to regulate inflammation in many diseases. Therefore, macrophages are potential pharmaceutical targets in many human and animal diseases. Although macrophages are capable of killing most of the microbes, many microorganisms (*Toxoplasma gondii*, *Leishmania sp*, *Mycobacterium tuberculosis *and *Listeria monocytogenes*) have developed potential ability to resist phagocytosis activity of macrophages. These pathogens subvert a macrophage's molecular machinery designed to kill them and come to reside in modified lysosomes. Therefore, nanoparticles-mediated delivery of antimicrobial agent(s) into pathogen-containing intracellular vacuoles in macrophages could be useful to eliminate cellular reservoirs [30,31]. This system can be used to achieve therapeutic drug concentrations in the vacuoles of infected macrophages and reduction in side effects associated with the drug administration and the release of pro-inflammatory cytokines. Polyalkylcyanoacrylates (PACA) nanoparticles have been used as a carrier for targeting antileishmanial drugs into macrophages. This nanomaterial did not induce interleukin-1 release by macrophages [32]. Therefore, similarly designed nanosytems could be very useful in targeting macrophage infections in chronic diseases.

The antifungal and anti-leishmanial agent amphotericin B (AmB) has been complexed with lipids-based nanotubes to develop a less toxic formulation of AmB. Gupta and Viyas [33] formulated AmB in trilaurin based nanosize lipid particles (emulsomes) stabilized by soya phosphatidylcholine as a new intravenous drug delivery system for macrophage targeting. Nanocarrier-mediated delivery of macrophage toxins has proved to be a powerful approach in getting rid of unwanted macrophages in gene therapy and other clinically relevant situations such as autoimmune blood disorders, T cell-mediated autoimmune diabetes, rheumatoid arthritis, spinal cord injury, sciatic nerve injury, and restenosis after angioplasty. Alternatively, nanoparticles with macrophage-lethal properties can also be exploited. Exploiting a variety of macrophage cell receptors as therapeutic targets may prove a better strategy for antigen delivery and targeting with particulate nanocarriers.

### Targeting inflammatory molecules

In the past two decades, many cell adhesion molecules have been discovered. Cell adhesion molecules are glycoproteins found on the cell surface that act as receptors for cell-to-cell and cell-to-extracellular matrix adhesion [34,35]. These cell adhesion molecules are divided into four classes called integrins, cadherins, selectins, and the immunoglobulin superfamily. These molecules are required for the efficient migration of inflammatory cells such as neutrophils and monocytes into inflamed organs and generation of host response to infections. There is, however, considerable evidence that excessive migration of neutrophils in inflamed lungs leads to exaggerated tissue damage and mortality. Therefore, a major effort is underway to fine tune the migration of neutrophils into inflamed organs. Recent advancements of the understanding of the cell adhesion molecules has impacted the design and development of drugs (i.e. peptide, proteins) for the potential treatment of cancer, heart and autoimmune diseases [36-38]. These molecules have important roles in diseases such as cancer [39,40], thrombosis [41,42] and autoimmune diseases such as type-1 diabetes [43-45]. The RGD peptides have been used to target integrins αvβ3 and αvβ5, and peptides derived from the intercellular adhesion molecule-1 (ICAM-1) have been used to target the αvβ2 integrin. Peptides derived from αvβ2 can target ICAM-1 expressing cells. Cyclic RGD peptides have been conjugated to paclitaxel (PTX-RGD) and doxorubicin (Dox-RGD4C) for improving the specific delivery of these drugs to tumor cells. Mice bearing human breast carcinoma cells (i.e., MDA-MB-435) survived the disease when treated with Dox-RGD4C, while all the untreated control mice died because of the disease [46]. This conjugate targets αvβ3 and αvβ5 integrins on the tumor vasculature during angiogenesis.

Extracellular regulated kinases (ERK) may regulate apoptosis and cell survival at multiple points that include increasing p53 and BAX action, increasing caspase-3 and caspase-8 activities, decreasing Akt activity, and increasing expression of TNF-α [47]. Our research group is investigating the interaction of RGD-RNT to αvβ3 integrins, following cell signaling through P38 kinases and its function in human lung epithelial cells, and bovine and Equine neutrophil migration. Cyclo(1,12)PenITDGEATDSGC peptide (cLABL peptide), derived from the I-domain of the α subunit of Leukocyte Function-Associated Factor-1 (LFA-1) is known to bind ICAM-1. cLABL peptide has been conjugated with methotrexate (MTX) to give MTX-cLABL conjugate [48]. Because ICAM-1 is upregulated during tissue inflammation and several different cancers, this conjugate may be useful for directing drugs to inflammatory and tumor cells. The anti-inflammatory activity of MTX is due to the suppression of production of anti-inflammatory cytokines such as (interleukin-6) IL-6 and (interleukin-8) IL-8. Thus, the activity of MTX-cLABL conjugate was compared to MTX in suppressing the production of these cytokines in human coronary artery endothelial cells stimulated with TNF-α. MTX-cLABL is more selective in suppressing the production of IL-6 than IL-8, which is opposite to MTX. PLGA nanoparticles coated with cLABL peptides have also been shown to upregulate ICAM-1 [49]. More detailed information on the mechanism(s) of internalization and intracellular trafficking of cell adhesion molecules is required to be exploited for delivering drug molecules to a specific cell type or for diagnosis of cancer and other diseases (heart and autoimmune diseases).

## Conclusion

It appears that nano drug delivery systems hold great potential to overcome some of the barriers to efficient targeting of cells and molecules in inflammation and cancer. There also is an exciting possibility to overcome problems of drug resistance in target cells and to facilitating movement of drugs across barriers such as those in the brain. The challenge, however, remains the precise characterization of molecular targets and to ensure that these molecules are expressed only in the targeted organs to prevent effects on healthy tissues. Secondly, it is important to understand the fate of the drugs once delivered to the nucleus and other sensitive cells organelles. Furthermore, because nanosystems increase efficiency of drug delivery, the doses may need recalibration. Nevertheless, the future remains exciting and wide open.

## Competing interests

The author(s) declare that they have no competing interests.

## Authors' contributions

Both the authors contributed equally in the preparation of this review article.
